# Advancing the Prediction and Understanding of Placebo Responses in Chronic Back Pain Using Large Language Models

**DOI:** 10.1002/ejp.70184

**Published:** 2025-12-12

**Authors:** Diogo A. P. Nunes, Dan Furrer, Sara Berger, Guillermo Cecchi, Joana Ferreira‐Gomes, Fani Neto, David Martins de Matos, A. Vania Apkarian, Paulo Branco

**Affiliations:** ^1^ Instituto de Engenharia de Sistemas e Computadores—Investigação e Desenvolvimento Lisbon Portugal; ^2^ Instituto Superior Técnico, Universidade de Lisboa Lisbon Portugal; ^3^ Department of Anesthesiology Northwestern University Feinberg School of Medicine Chicago Illinois USA; ^4^ Center for Translational Pain Research, Northwestern University Feinberg School of Medicine Chicago Illinois USA; ^5^ Department of Neuroscience Northwestern University Feinberg School of Medicine Chicago Illinois USA; ^6^ Responsible and Inclusive Technology (Exploratory Sciences Division), IBM Research Yorktown Heights New York USA; ^7^ Computational Psychiatry and Digital Health (Impact Science Division), IBM Research Yorktown Heights New York USA; ^8^ Departmento de Biomedicina, Unidade de Biologia Experimental, Centro de Investigação Médica (CIM), Faculdade de Medicina Universidade do Porto Porto Portugal; ^9^ i3S—Instituto de Investigação e Inovação Em Saúde University of Porto Porto Portugal

**Keywords:** chronic pain, language, large language models, machine learning, placebo

## Abstract

**Background:**

Placebo analgesia is a widely studied clinical phenomenon, yet placebo responses vary widely across individuals. Prior research has identified biopsychosocial factors that determine the likelihood of an individual to respond to placebo, yet generalizability and ecological validity in those studies have been limited due to the inability to account for dynamic personal and treatment effects.

**Methods:**

We assessed fine‐tuned large language models (LLMs) for the prediction of placebo responses in chronic low‐back pain using contextual features extracted from patient interviews, as they speak about their lifestyle, pain, and treatment history. Interviews were conducted as part of two RCTs designed to study the placebo effect. These interviews were collected after treatment in the first trial (discovery cohort) and prior to treatment in the second trial (validation cohort).

**Results:**

Semantic features extracted with LLMs can predict which individuals respond to a placebo, with an accuracy of 74% in unseen data, and validating with 70% accuracy in an independent cohort. Furthermore, in contrast to previous work, LLMs eliminated the need for pre‐selecting search terms, enabling a fully data‐driven approach, and provided interpretable insights into psychosocial factors underlying placebo responses.

**Conclusions:**

These findings expand on prior research by integrating state‐of‐art NLP techniques to address limitations in interpretability and context sensitivity of the traditional methods in related work. This method highlights the role of language models to link language and psychological states, paving the way for a deeper quantitative exploration of biopsychosocial phenomena, and to understand how they relate to treatment outcomes.

**Significance Statement:**

This study paves the way for a deeper yet quantitative exploration of biopsychosocial phenomena through language, and to understand how they relate to treatment outcomes, namely placebo. In this case it highlights nuanced linguistic patterns linked to responder status, which tap into semantic dimensions such as “anxiety,” “resignation,” and “hope”.

## 1 Introduction

The placebo effect is a widely studied clinical phenomenon, by which an inert substance can strongly influence psychological and physiological states. Placebo responses are notably large and frequent in chronic pain treatments (van Lennep et al. 2021). Harnessing placebo for pain management offers opportunities for non‐pharmacological, non‐opioid treatments; however, strong placebo responses can obscure drug effects and complicate clinical trial design for developing analgesic drugs (Finnerup et al. 2018; Kaptchuk et al. 2020; Tuttle et al. 2015; Vase and Wartolowska 2019). Placebo responses are thought to be driven by biological, contextual, and affective cues (Atlas 2021), but not everyone responds equally to placebos (Vachon‐Presseau et al. 2018). Thus, there is an impetus to discover the specific traits that make an individual more likely to respond to placebo. Identifying such traits and being able to predict placebo responders could have strong implications for patient triage in clinical trials, as well as in pain management and patient care.

Previous studies have demonstrated that both neurophysiological factors (e.g., brain properties) and personality factors (e.g., interoceptive awareness) can predict placebo responses (Vachon‐Presseau et al. 2022; Vachon‐Presseau et al. 2018), but with weak generalizability and limited ecological validity. Since placebo responses are thought to be driven by biopsychosocial aspects, it is unclear whether relatively stable brain properties or personality traits can capture nuances in the multiple contexts in which a patient engages with the treatment. Indeed, the patient's situational assessment of a treatment outcome is dynamic, changing in response to prior treatment experiences, coping strategies, identity, and a multitude of other psychosocial factors. Placebo responses have been shown to be impacted by these contextual contributions (Atlas 2021; Horing et al. 2014), and thus novel approaches accounting for them are required. This contextual information is most readily accessible through the analysis and quantification of natural language. Indeed, in the context of a randomised‐controlled trial (RCT) in chronic low‐back pain, we have previously shown that such features can be quantified using natural language processing (NLP), e.g., through semantic proximity to topics such as fear and stigma, and that analgesic responses to placebo can be predicted with high accuracy (79%) from post‐treatment interview language (Berger et al. 2021; Branco, Berger, et al. 2023). These results were later validated with an independent cohort using pre‐treatment interviews, showcasing the generalizability of the method and its ability to predict placebo responses a priori (Branco, Berger, et al. 2023).

Recent advancements in NLP have significantly improved the ability to capture psychosocial traits from language. Pretrained Large Language Models (LLMs) learned to encode linguistic knowledge from large amounts of diverse language data and can be fine‐tuned to learn concepts and associations from specific domains, such as pain. When analyzing a given word, LLMs enforce the observation of neighboring words, embedding this context into its perceived meaning (Vaswani et al. 2017). These models thus allow for extracting domain‐adapted, rich semantic representations that can be leveraged for developing and interpreting a placebo response predictive model, overcoming the limitations of past work. In this study, we test our ability to predict and interpret placebo responses by developing a new predictive model based on LLMs. We leverage social media data to train LLMs in the pain domain and re‐analyze interview data from two RCTs for chronic pain, designed to identify which individuals are more likely to show pain relief after receiving a placebo. In the first trial, interviews were conducted after treatment intervention; we use these data for model development. In the second trial, interviews were conducted prior to treatment intervention, mimicking the predictive nature of our task; we use these data as an independent source of model validation. We further leverage these data to develop an interpretability pipeline that uncovers which features increase the likelihood of a placebo response.

## 2 Methods

This study reports a re‐analysis of data from two RCTs investigating predictors for placebo in chronic low‐back pain (CBP) patients [ClinicalTrials.gov registration ID: NCT02013427]. Details of these trials were published previously (Vachon‐Presseau et al. 2022; Vachon‐Presseau et al. 2018), and are presented summarily here for brevity. They included two double‐blinded independent studies in CBP patients, reporting pain greater than 4/10 on a visual analog scale, a history of CBP for at least 6 months prior to enrollment, no pain at other body sites, and no comorbid neurological or psychological disorders. Participants were instructed to discontinue any ongoing pain medication (largely over‐the‐counter NSAIDs) after enrolling in the RCT. The first study was designed to build predictive models of placebo response, and the second study was designed to validate the best model from the first study in an independent sample/study. The two studies were conducted approximately 2 years apart, and both the patients and research staff enrolled in each study were different to enhance generalizability. Both studies were approved by Northwestern University's Institutional Review Board, and all participants signed a consent form.

**FIGURE 1:**
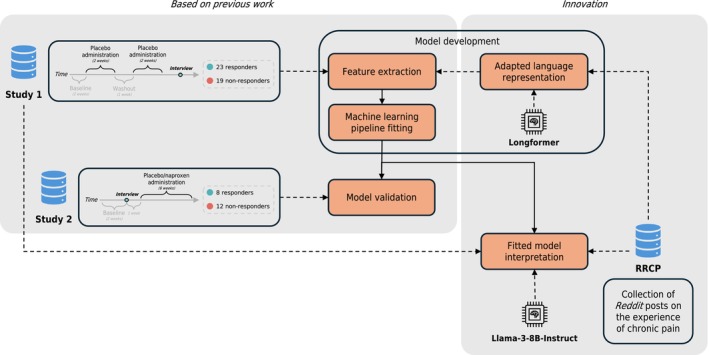
Study design. Textual data from RRCP are used to continually pretrain (i.e., fine‐tune) the Longformer language model for pain‐domain adaptation. Textual data from study 1 are used for feature extraction with the adapted Longformer and fitting the machine learning pipeline for the placebo response prediction task. This fitted model is interpreted using a variety of semantics, namely from study 1 and RRCP, leveraging the Llama‐3‐8B‐Instruct LLM. This model is also validated on textual data from study 2.

These data were previously used to build predictive placebo models (Berger et al. 2021; Branco, Berger, et al. 2023) via conventional language modelling techniques including dictionary‐based approaches (Tausczik and Pennebaker 2009) and latent semantic analyses (Deerwester et al. 1990), which extract semantic vector‐based representations (also called embeddings) for each word independently of the surrounding text, i.e., context. Here, we build upon this previous research by developing, validating, and interpreting a model predictive of a patient's placebo treatment response using LLMs, taking advantage of contextual features extracted from their interview—that is, linguistic features which capture the meaning of a word based on the word itself and neighbouring words—and further using LLMs to gain novel insights into the learned model (Figure 1). This context‐sensitive feature extraction is especially important, and an improvement over past work, for language classification tasks where the meaning of a word can change depending on its context of use, effectively tackling challenges related to polysemy and the use of metaphors, as is frequent in the verbal expression of pain (Halliday 1998; Lascaratou 2007; Malani 2010), and correctly assigning different meanings to repeated words when surrounded by different contexts. Additionally, words such as the pronoun “it” can be enriched with surrounding meanings, instead of being represented by a fixed embedding vector or removed as a standard stop word, disrupting the natural structure of the text (Siino et al. 2024; Vaswani et al. 2017). Thus, contextual embeddings have the potential to produce richer representations of the text, potentially leading to better predictive performance and significantly improving the interpretability of results when coupled with explainable artificial intelligence. The code for all analyses is available at: https://gitlab.hlt.inesc‐id.pt/cylons/pain/placebo‐public‐code.

### 2.1 Participants, Data, and Study Design

#### 2.1.1 Study 1 Participants and Clinical Trial Design

In the first study (Berger et al. 2021; Vachon‐Presseau et al. 2018), 125 participants with CBP were enrolled and 66 completed all aspects of the study, including the language interviews. Full demographic data for study 1 can be found in the Data S1. Participants were randomly assigned to one of two arms: treatment (*N* = 46) and no‐treatment (*N* = 20). Patients in the treatment group were further split into active treatment (*N* = 4, Naproxen, 500 mg + Esomeprazole, 20 mg) and placebo treatment (*N* = 42, 2 lactose pills) subgroups. The size imbalance between active and placebo treatment subgroups was intended for blinding purposes only, given that the study was designed to study placebo responses. This way, investigators could ethically tell patients they might receive a drug or a placebo without overt deception. The allocation odds were not disclosed to the participants. In this study we use data only from the placebo treatment arm. We also used no treatment data for the fine‐tuning of the language model (see below) and post hoc to ensure model predictions could not explain pain changes in this group, thus ruling out treatment effects and regression to the mean and natural history effects. Prior to treatment commencement, pain ratings were obtained twice daily over 2 weeks via a smartphone app to establish baseline pain. This long baseline period was designed to reduce concerns about regression to the mean or natural recovery (as fluctuations during this period are already incorporated into the definition of placebo response, see also below). Afterwards, subjects completed 2 two‐week treatment periods followed by a one‐week washout. Throughout treatment and washout periods, participants continued to rate their pain twice daily. Treatment outcomes were measured during the last week of the treatment phase. To identify individuals who exhibited significant placebo‐induced analgesia, a permutation t‐test was applied to each participant's pain ratings, testing whether ratings during the treatment phase were lower than at baseline. If pain intensities for either of the treatment phases (i.e., twice a day over 14 days = 28 pain ratings) were statistically lower than baseline (also 28 pain ratings), patients were labelled placebo responders. Otherwise, they were labelled placebo non‐responders. We note that this method provides a statistical method to define significant pain relief, without relying on arbitrary cutoffs. We also note that here we classify subjects as ‘placebo responders’ to refer to participants predicted to show a placebo response in this specific context and study, not to imply a stable responder phenotype; the current study design, with only one interview, does not allow us to identify responder phenotypes.

After the treatment and washout periods, all participants were interviewed regarding their personal experience of pain. The interview followed a semi‐structured script with prompts covering 16 topics of interest (27.2 ± 10.3 min) but was open‐ended such that the sequence of topics followed the natural flow of the conversation (see Data S1 and the original article (Berger et al. 2021) for more details).

#### 2.1.2 Study 2 Participants and Clinical Trial Design

The second study (Branco, Berger, et al. 2023; Vachon‐Presseau et al. 2022) enrolled 94 CBP participants, out of which 50 completed all aspects of the study. 8 participants were randomised to a no‐treatment arm and 42 to the treatment arm. In this study, however, participants were *equally* randomised to an active group (*N* = 22, Naproxen, 500 mg + Esomeprazole, 20 mg) and a placebo group (*N* = 20, 2 lactose pills). This was to investigate effect specificity and the additive nature of placebo effects over drug effects. Full demographic data for study 2 can be found in the Data S1. As in study 1, placebo responders and non‐responders were classified by permuting pain ratings from 2 weeks prior to treatment compared to the single treatment period (6 weeks). However, unlike study 1, study 2 participants were interviewed about their pain experience at the start of the study (before submitting baseline ratings), such that any predictive ability could be dissociated from treatment effects. Since study 2 was designed to replicate findings from study 1, the study's interview script was considerably smaller than study 1 (3.27 ± 1.67 min), covering 4 of the original 16 topics, based on the topics that provided the most predictive signal (see Data S1 and the original article (Berger et al. 2021) for more details).

#### 2.1.3 Reddit Reports of Chronic Pain (RRCP)

Besides study 1 and study 2 data, we also used data collected from social media to fine‐tune our feature extraction language model and assist with interpretability (see below). Social media, and Reddit in particular, has been increasingly used to learn the relation between real‐world language and health (Almeida et al. 2024; Cummins et al. 2025; Dan et al. 2025). RRCP (Nunes et al. 2023) is a dataset composed of Reddit posts, scraped from the web, on select subreddits (i.e., self‐moderated sub‐forums focusing on a specific topic). One of the included subreddits is “r/backpain,” which focuses discussion on the topic of back pain, our studied clinical population. For this subreddit, RRCP contains 2667 individual posts (from its inception up to 2020). Details about demographic, geographic, and text sentiment distributions of these posts can be found in the original paper (Berger et al. 2021). According to Reddit's Data API terms, academic research on the platform's public content is permitted (Usage 2024). We did not attempt to identify or contact any Reddit user, and we do not report any identifying information. We do not publicly share any models trained on Reddit data, according to Reddit's Data API terms.

### 2.2 Preprocessing, Data Cleaning, and Feature Extraction

After interview transcription and quality control, the text was preprocessed, and semantic (contextual) features were extracted. For study 1, since the interview covered 16 topics in various temporal orders, each subject's answer was automatically annotated for each of the topics, and then visually inspected and corrected for accuracy. All text was preprocessed with the same pipeline: (1) replacing all transcription annotations, e.g., “(inaudible at timestamp),” Reddit tags (subreddit and user), and URLs, with the “[UNK]” token (standing for “unknown”; a token is an instantiation of a vocabulary term); and (2) stripping all HTML tags, markdown styling, emojis, and multiple whitespaces. We maintained the original capitalization of all text. For study 1 only, we removed the last two topics from all interviews due to their close relation to the study itself and consequent limited generalizability (opinion of the study and other miscellaneous topics), and the study medication topic for the same reasons, along with the fact that participants in the no‐treatment arm did not answer this question. We did not remove any topic from study 2 interviews. We considered only the main body of text in all Reddit posts.

Once the text was preprocessed, segmented, and annotated, we extracted semantic representations (i.e., contextual feature vectors, or embeddings) for each interview from a language model, in this case, the publicly available pretrained Longformer language model (Beltagy et al. 2020), fine‐tuned on RRCP text (i.e., domain adaptation, detailed below). We chose this model for several reasons: it is open source; it allows the processing of larger text sequences (i.e., 4096 tokens), as opposed to other similar models, such as BERT (Devlin et al. 2019) and RoBERTa (Liu 2019) (i.e., 512 tokens), thus making it more adequate for long interviews (study 1: 3366.36 ± 1768.29 tokens, study 2: 450.05 ± 330.07 tokens); and it has been shown to represent long sequences of text in useful ways for various tasks, such as question answering and document classification (Beltagy et al. 2020). We fine‐tune Longformer on RRCP text, since the base model is trained on large‐scale generic text and is thus domain‐agnostic and possibly lacking in knowledge about pain‐specific topics. We also report results using the base Longformer model for completeness.

Because interviews from both study 1 and 2 are obtained through specific topic questions, we also compared the impact of text structuring on contextual feature extraction. Thus, we structured interviews in two ways. The first, referenced as “sequential text,” assumes the original transcript sequence and represents the interview as a single document (a document is a single input sequence of text for a given patient). The second, referenced as “topic groupings” concatenates all segments of the same topic in each interview, effectively representing a single interview as a set of documents, each focusing on a single topic. Note that this approach does not change the text content; it re‐arranges the text sequence based on the topic sequence such that it follows the same order across participants. In other words, while the former represents the text in line with the flow of the conversation regardless of topic coherence, the latter represents the text based on semantic information that is topic specific.

### 2.3 Definition of the Document Feature Vector

The Longformer model outputs a 768‐dimensional vector for each token in the input document. We defined the document feature vector as the pooling of all token embeddings, summarising latent semantic dimensions across the whole document. We compared two of the most common strategies to aggregate multiple vectors: mean and max pooling (Chen et al. 2018; Lascaratou 2007; Reimers 2019). Mean pooling calculates the mathematical average of all token vectors. This approach likely dilutes sparse or extreme signals and thus reflects the overall (i.e., mean) semantic content of the whole document. This averaging potentially increases the similarity of the semantic representation vectors across subjects and therefore may generalise better, but at the cost of also averaging out predictive signal. In contrast, max pooling attributes the maximum value observed across tokens for each vector element, effectively identifying the most salient attributes of each document. This approach will identify signals in a more specific way for each interview and may be able to pick up on unique predictive signals, at the cost of being less able to identify common language patterns across subjects, lending itself to potentially weaker generalizability.

**FIGURE 2:**
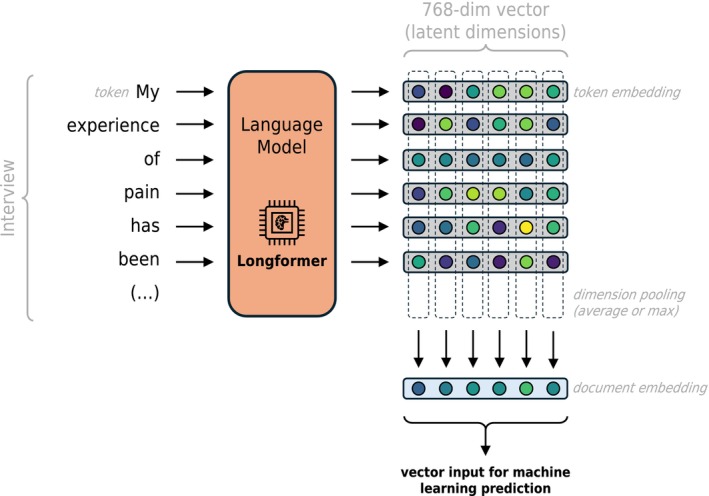
Contextual feature extraction. A single patient interview may be formatted as a single document, or a list of documents. A document is a list of tokens (as a simplification, a token may be understood as a single word). This document is given as input to the Language Model (Longformer), which produces a 768‐dimensional vector for each token (also called the token embedding). The architecture of the Language Model is such that each individual token embedding is informed by the remaining tokens in the document, hence producing a contextual feature vector. The input document is represented by a single 768‐dimensional vector (also called the document embedding) by pooling (i.e., aggregating) the individual token embeddings across each dimension. The document embedding for a given patient's interview is that patient's contextual feature vector.

Longformer is designed for long input documents, up to 4096 tokens. However, 26% of study 1 interviews are over this limit. To avoid truncating the original text arbitrarily at the maximum length mark, for these specific cases, the tokenized document was split into two overlapping windows of 4096 tokens each, before feature extraction. Note that, in these cases, the resulting document feature vector was calculated by pooling its left and right window vectors, matching the token mean or max pooling strategy. The overlap allowed the two windows to share some of their context (i.e., neighbouring words) (Figure 2).

### 2.4 Fine‐Tuning of Longformer

The pretrained Longformer model provides domain‐agnostic contextual feature vectors. This means that textual documents are projected into an n‐dimensional space, such that vector similarities encode generic syntactic and semantic similarities between multiple documents. Since pain interviews employ specific morphosyntactic patterns and metaphoric references (Halliday 1998; Lascaratou 2007; Malani 2010), there is reason to believe that biasing the language model towards the language of pain would result in more useful feature vectors (for the pain domain); intuitively, the qualifiers “sharp” and “numb” are contextually sound in the pain domain, but not necessarily so in other domains—thus, allowing the encoding model to learn the language of pain from a real‐world source could significantly improve its language representation capacity. This and similar techniques have been shown to improve predictive performance in similar tasks (Cossu et al. 2024). To this end, we fine‐tune the Longformer model on the Masked Language Modelling (MLM) task with domain‐specific text, obtained from the RRCP dataset (also known as domain adaptation). MLM is a self‐supervised learning task, meaning that the supervisory signals are automatically and randomly generated from the input data itself (Devlin et al. 2019). MLM, particularly, is a common language modelling task that forces the model to learn syntactic and semantic relations between vocabulary tokens by having the model predict randomly masked tokens in the input text; indeed, Longformer was pretrained on MLM with a diverse and multi‐domain corpus (Beltagy et al. 2020). For model fine‐tuning to be successful, it requires that the training data is curated to be domain‐specific, such that the model learns our specific domain of pain, and a clear stopping criteria (number of training steps and parameters) to ensure the model stops fine‐tuning before it starts losing pretrained knowledge, i.e., when the model starts overfitting to training data and forgets what it had learned during pretraining, also known as catastrophic forgetting (McCloskey and Cohen 1989).

To address the domain specificity of RRCP texts, since our patient sample contained only patients suffering from chronic back pain, we limited the MLM fine‐tuning textual data to posts on the “r/backpain” subreddit. We further automatically filtered these posts to those discussing relevant topics of interest, according to the interview topics of study 1 (see Data S1 for more details on the filtering algorithm). We deemed this filtering necessary to further curate the selection of RRCP texts, as we observed in‐domain but out‐of‐topic posts, such as advertisements for websites or tools to deal with chronic back pain, and recruitment calls for back pain studies. A total of 1893 posts (out of 2667) from the “r/backpain” subreddit were used for MLM fine‐tuning.

To control the amount of fine‐tuning, we defined three loss functions, measured at every 50 training steps (see Data S1 for more details). These measured how well the model predicted each masked token on three distinct validation datasets: specifically, 10% of the posts selected from “r/backpain” (190 documents), study 1 interviews from patients in the no‐treatment group (20 documents; not used in the placebo treatment prediction task), and 10% of the test subset of the publicly available WikiText dataset (wikitet‐2‐v1; 436 documents; (Merity et al. 2016)). We expected the minimization of the loss on each of the three datasets to reflect, respectively, the desired biasing towards the pain domain, the approximation to the clinical interviews used for the predictive model development, and the retaining of the pretrained knowledge. We also used the number of frozen layers as an additional optimization parameter, balancing retaining pretrained knowledge whilst approximating the pain domain. We chose the fine‐tuned checkpoint of Longformer that most minimized all three loss functions.

### 2.5 Machine Learning Pipeline

The machine learning pipeline was selected to be identical to the best performing pipeline of our original study (Berger et al. 2021), such that current performance could be benchmarked. Specifically, it included feature scaling and selection, and a Linear Support Vector Classifier (LinearSVC) model with L1 regularisation. All base models were obtained from sklearn (Pedregosa et al. 2011). Feature scaling and selection leveraged the RobustScaler and SelectKBest implementations, respectively. Our original study included additional variations of models and regularisation (Berger et al. 2021), for which we also present results, for completeness, in the Data S1.

This pipeline was fitted to study 1 feature through nested cross‐validation (CV). The inner CV, implemented with sklearn's GridSearchCV, performed hyperparameter optimization in 10 stratified folds, specifically searching for the best combination of the number of features to select (parameter k in SelectKBest) and the amount of model regularization (parameter C in LinearSVC). The outer CV, implemented with Leave‐One‐Out CV, fitted and tested (with the one left‐out sample) the machine learning pipeline with the optimized hyperparameter configuration, ensuring proper independence between training and validation samples. We measured performance based on accuracy score, since the data is well balanced. We compared performance against the null distribution by permuting the labels and assessing the likelihood of the prediction accuracy occurring by chance. We tested models for four different iterations, as mentioned above: (1) mean pooling and sequential text; (2) max pooling and sequential text; (3) mean pooling and topic grouping; and (4) max pooling and topic grouping.

As in our previous study (Branco, Berger, et al. 2023) the best‐performing model (from the four iterations described above) was independently validated using data from study 2. To do so, the best performing model was refit in study 1 and validated on study 2. A key methodological challenge here lies in the differences between the two studies: study 2 interviews were shorter and covered only 4 of the 16 topics assessed in study 1. In our earlier work (Branco, Berger, et al. 2023), a model trained on study 1 data validated successfully on study 2 data, despite differences in interview length. This was likely possible due to the methodology (i.e., averaging signals across all tokens in the interview), capturing broad themes more amenable to generalisation. Here, however, the methods are more context‐sensitive, and predictive features may depend on information not available in study 2 interviews. To address this, we implemented two separate validation approaches: first, we applied the model “as‐is” to study 2; second, we retrained the best performing model only for the 4 overlapping questions in study 1, allowing for hyperparameters to be tuned, and validated this model with study 2. We did not test any other models on the validation dataset. Results from both approaches are reported.

### 2.6 Interpretation

One major disadvantage of deep learning feature vectors (e.g., the 768‐dimensional contextual feature vectors used in our machine learning pipeline) is the difficulty in interpreting what kind of semantic or linguistic signal each dimension is capturing, if not multiple signals at once. This lack of real‐world anchors hinders model interpretability and the acquisition of new knowledge about the underlying task. Input perturbation methods, such as LIME (Ribeiro et al. 2016) and SHAP (Lundberg 2017), target these challenges but fall short of providing novel, concrete, and actionable knowledge about the underlying task. In this work, we adopted two approaches to interpreting machine learning selected features. The first interpretation approach assessed associations between the extracted language model features and previously established placebo predictive features and linguistic variables—these are detailed below. The second interpretation approach focused on further exploring the semantic space used by the fitted model, understanding the fitted decision boundary that separated placebo responders from non‐responders and which semantic concepts lay on either side of that boundary. This has the potential to uncover concepts that the fitted model deemed “placebo responder”‐like and “placebo‐non‐responder”‐like but may not have otherwise been seen in correlational or qualitative thematic approaches.

#### 2.6.1 Associations With Psychological and Demographic Features

To begin interpreting the contextual features captured by Longformer and later selected by the machine learning pipeline, we correlated each of these features with linguistic and demographic variables, clinical questionnaires, and psycholinguistic assessments, which had been explored in previous works (Berger et al. 2021; Branco, Berger, et al. 2023; Vachon‐Presseau et al. 2022; Vachon‐Presseau et al. 2018). We first investigated whether the features were associated with patient age, verbosity (i.e., the number of words in the interview), and vocabulary (i.e., the number of unique words in the interview), to ensure our predictions were not driven by lower‐level linguistic information. Then, we assessed if the features were associated with other psychological features shown to predict placebo in our previous work. To do so, we selected relevant clinical questionnaire subfields, namely “openness” (from the big 5 personality dimensions), and 4 subscales from the Multidimensional Assessment of Interoceptive Awareness (MAIA): “Not Distracting” (MAIA/nd), “Attentive Regulation” (MAIA/a), “Emotional Awareness” (MAIA/e), and “Self‐Regulation” (MAIA/sr). These were identified by Vachon‐Presseau et al. (2018) as the features most correlated to the magnitude of placebo response. Finally, we studied the relationship between current features and prior semantic features; this included psycholinguistic measures like “drives”, “achievement”, and “leisure” from Linguistic Inquiry and Word Count (LIWC) (Tausczik and Pennebaker 2009), as well as semantic distance (SD) measurements to target topics, namely, “magnify”, “afraid”, “fear”, “awareness”, “loss”, “identity”, “stigma”, and “force”. These were identified by Berger et al. (2021) as the most significant features for placebo treatment response classification in their work.

#### 2.6.2 Exploration of Semantic Concepts Within the Contextual Feature Space

The machine learning pipeline described above, once fitted to study 1 interviews, defines a hyperplane (i.e., decision boundary) separating the target classes in the contextual feature space. The farther a data point is from this boundary, the more confident the model is in its prediction (positive or negative). Because the contextual feature space encodes latent semantics in any given string of text, one possible approach to interpret that space and the decision boundary is to project a series of curated concepts (each represented by one or multiple strings of text) and measure their distance to that boundary. In other words, new strings of text can be passed through the language model feature extractor—producing semantic feature vectors—which can then be projected to our decision space and inform how a given concept is interpreted by our classification model. The farther a concept (e.g., “scepticism”) is from the decision boundary, the more confident the machine learning pipeline is in its being “placebo‐responder”‐like or “placebo‐non‐responder”‐like. Distances greater than zero classified concepts related to placebo responders, whilst distances smaller than zero classified concepts related to placebo non‐responders.

We defined the set of concepts (or semantics) in three ways. First, we assessed how concepts hypothesized to be related to placebo would map onto our semantic space. To do so, we selected a set of 261 a priori defined words, such as “wonder,” “anxiety,” “resilient,” and “hope,” that were used as predictive features in our original study (Berger et al. 2021)—these words, drawn from ethnographies, questionnaires, and peer‐reviewed studies, were hypothesized to reflect chronic pain experiences and placebo qualities (see Berger et al. 2021 for details). Second, to explore topics used by the participants without restricting them to pre‐defined words, we defined the second set of semantics according to study 1 interview topics. To this end, we extracted all individual sentences from study 1 interviews, their corresponding feature vectors, and clustered them in the feature space, using HDBSCAN (Campello et al. 2013), a hierarchical unsupervised clustering algorithm. We expected individual sentences to most likely focus on a single latent concept and be more contextualized than individual words (e.g., “anxiety” versus “I really had no anxiety,” or “my anxiety was through the roof”). We leveraged hierarchical unsupervised clustering in two ways: (1) to group all sentences focusing on the same or similar latent concept, defining a unit of semantics, and (2) to discard all individual sentences that could not be clustered according to the algorithm, filtering the analyzed semantics only to those most discussed (curation). Thus, the second set of semantics was defined as the list of sentence clusters found in study 1. We prompted Llama‐3‐8B‐Instruct (Dubey et al. 2024), a publicly available LLM, to label each cluster of sentences with a short description (see Data S1 for prompting details), for ease of interpretation of the latent concept represented by the corresponding cluster of sentences. LLMs have been noted for their summarization capabilities and have further shown that model prompting is the key for better results in similar tasks, as opposed to model size, justifying the use of a cheaper and “smaller” 8 billion parameter model (Zhang et al. 2024). Notably, although not fixed a priori, the scope of this second set of semantics was limited by study 1 interview topics. Third, we wanted to add chronic pain text concepts from novel, unconstrained text to further help understand the model's boundaries. To do so, we performed the same clustering method as mentioned above but with the list of sentences extracted from “r/backpain,” from RRCP. In this case, the semantics were determined and controlled independently of this work, which we expected to provide novel insights into what semantic properties the model was using to classify placebo responders vs. non‐responders.

## 3 Results

### 3.1 Study 1 and 2 Samples and Placebo Responses

In the first study, out of the 42 patients with complete data, 23 patients (~55%) reported significant pain relief (statistically significant change from baseline) and were classified as placebo responders, while 19 patients (~45%) did not experience such relief and were classified as non‐responders. In the second study, out of the 20 participants receiving a placebo pill, 8 (~43%) were classified as responders and 12 (~57%) were non‐responders.

### 3.2 Placebo Response Prediction

This study included a comparison of the pooling strategies to obtain document contextual feature vectors from token vectors (as outputted by the fine‐tuned Longformer)–mean vs. max pooling–, and the comparison of text representation strategies –sequential vs. by topic–, for a total of four feature extraction configurations. For conciseness, here we describe only the results with the mean pooling strategy and sequential text representation, since this was the best performing configuration. We also present the results for the same experiment configuration using contextual features from the baseline pretrained Longformer model (i.e., not fine‐tuned with RRCP text). The results for all other feature extraction configurations and alternative machine learning pipelines are shown in Data S1.

**FIGURE 3:**
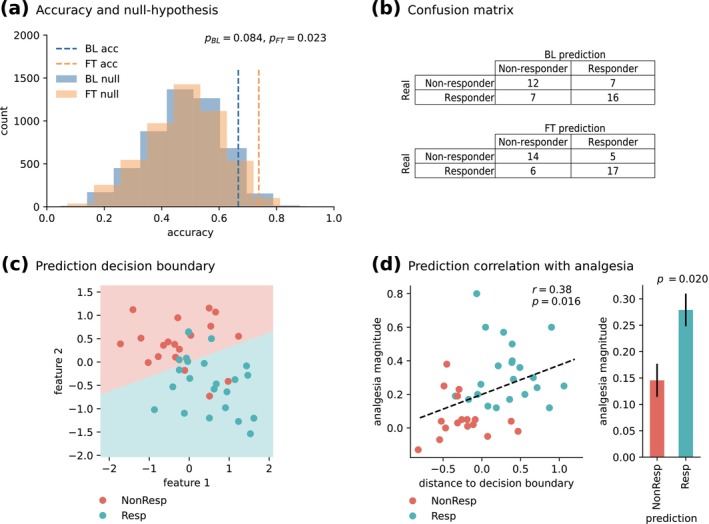
Predictive model performance. (a) Outer cross‐validation accuracy based on the baseline (BL) and fine‐tuned (FT) Longformer models for feature extraction, for the mean pooling and sequential text representation experiment configuration. Both results are shown in comparison to the corresponding null hypothesis distributions. The BL approach had a non‐statistically significant accuracy of 67% (*p* = 0.084), whilst the FT approach had a statistically significant accuracy of 74% (*p* = 0.023). (b) The corresponding outer cross‐validation confusion matrices. (c) Fitted decision boundary, in the selected two‐feature space, in relation to the actual study 1 patient labels. Background colours represent the learned class separation, whilst the dots on top represent the actual study 1 placebo treatment subgroup patients. Red dots over red background and blue dots over blue background represent accurate predictions. (d) Relation between study 1 placebo treatment predictions and analgesia magnitude. On the left scatter plot, the analgesia magnitude is significantly positively correlated (*r* = 0.38; *p* = 0.016) with the distance to the decision boundary (i.e., confidence in the prediction), as shown in panel A. The right bar plot shows the expected analgesia magnitude of predicted placebo treatment outcomes. The difference between the two groups is statistically significant (*p* = 0.020).

With the mean pooling strategy and sequential text representation, our model achieved statistically significant accuracies of 72% and 74% in the inner and outer CV, respectively (outer CV: *p* = 0.023, bootstrapped 95% CI [60, 93]). Figure 3a shows these results in relation to the null‐hypothesis distribution, and Figure 3b shows the corresponding outer CV (Leave‐One‐Out) confusion matrix. Note that Figure 3a shows the same metrics for the baseline Longformer model, without any fine‐tuning with RRCP text, for comparison (non‐statistically significant accuracy of 67% on the outer CV: *p* = 0.084, bootstrapped 95% CI [62, 93]), providing further evidence for the hypothesized need for fine‐tuned domain adaptation of the contextual feature extraction method. Due to the overlapping confidence intervals, we cannot conclude that the domain‐adapted model is statistically superior to the baseline model. However, the domain‐adapted model does outperform the baseline when compared against the null distribution.

### 3.3 Relation Between Prediction and Analgesia

For model interpretation, we took the mean pooling and sequential text representation experiment configuration and observed the parameters of the machine learning pipeline fitted on all study 1 interviews (with hyperparameter optimization). This model had 72% accuracy in the 10‐fold stratified GridSearchCV, and 79% accuracy on all training samples. Moreover, the machine learning pipeline feature selection step discarded all but two contextual features, out of the 768‐dimensional feature space, which we reference as feature 1 and feature 2. Figure 3c shows the model's fitted decision boundary in relation to features 1 and 2, as well as study 1 interviews and their true labels (dots). In this panel, red dots over red background (placebo non‐responders) and blue dots over blue background (placebo responders) represent accurate predictions. Also, the farther a dot is from the decision boundary, the more confident the prediction. Figure 3d shows a statistically significant positive correlation between this distance and the actual analgesia magnitude (*r* = 0.38; *p* = 0.016), as well as the expected average analgesia magnitude of study 1 placebo patients predicted as responders (27.9% pain relief) and non‐responders (14.6% pain relief). The difference in analgesia magnitude between these two groups was statistically significant (independent samples *t*‐test, *p* = 0.020, Cohen's *d* = 0.68); in other words, individuals predicted as placebo responders did, indeed, show significantly higher levels of pain relief.

This model's predictions over study 1 participants in the no‐treatment arm do not significantly correlate with their analgesia magnitude (*r* = −0.023, *p* = 0.922), and there is no statistically significant difference in the expected average analgesia magnitude of the no‐treatment arm participants predicted as responders and non‐responders (independent samples *t*‐test, *p* = 0.597, Cohen's *d* = 0.11), thus pointing to the specificity of placebo effects in our predictions, instead of spontaneous recovery or regression to the mean. Furthermore, features 1 and 2 were not statistically different between participants in the treatment arm versus the placebo arm (feature 1: independent samples *t*‐test, *p* = 0.768, Cohen's *d* = 0.08; feature 2: independent samples *t*‐test, *p* = 0.985, Cohen's *d* = 0.005), also ruling out that the model is picking up on treatment effects, despite the interview being conducted after the treatment phase.

### 3.4 Anchoring of Contextual Features on Psychological and Demographic Features

**FIGURE 4:**
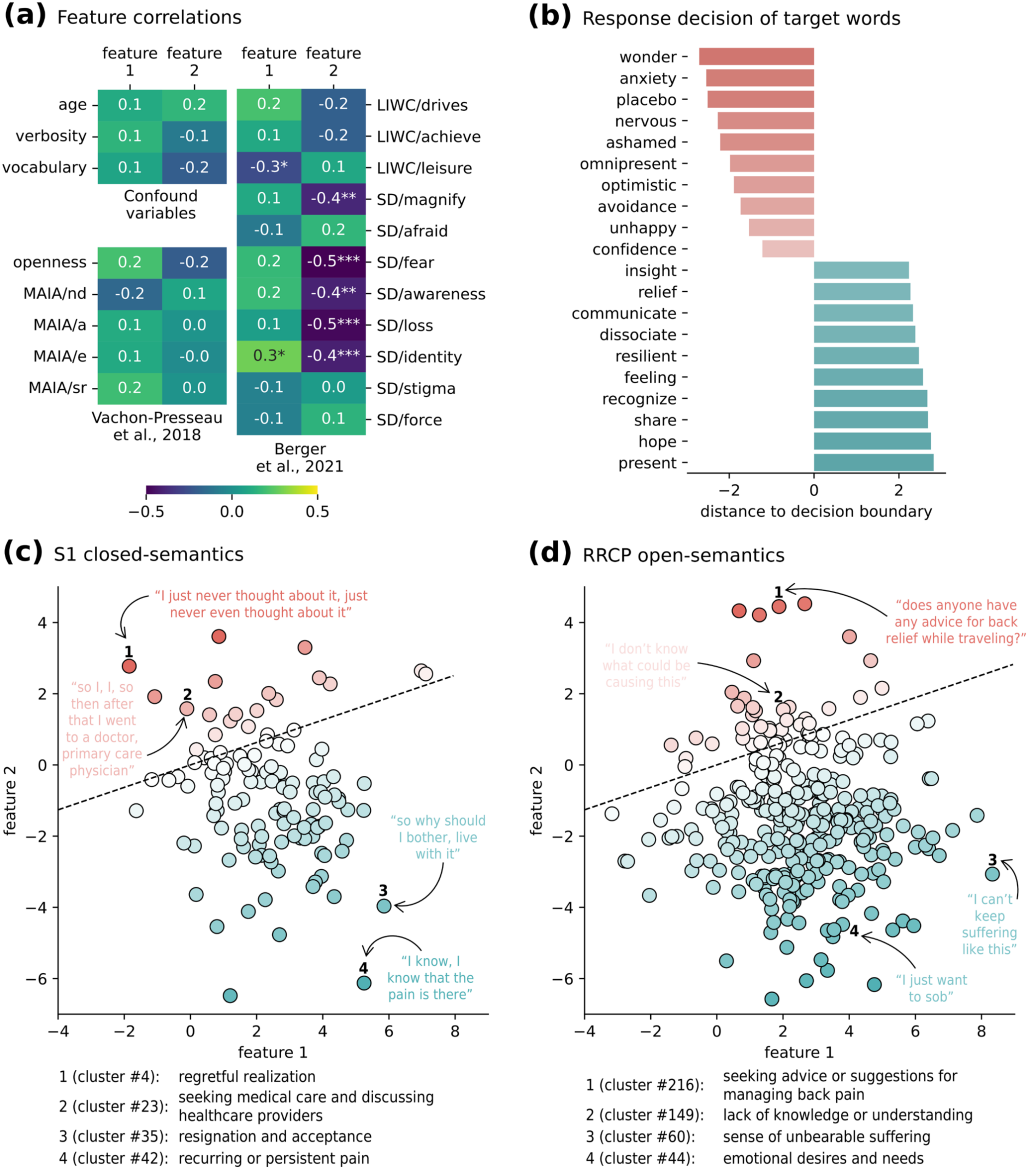
Predictive model interpretation. (a) Pearson correlation between the two selected contextual features (identified as feature 1 and 2) and other variables of interest; *p < 0.05, **p < 0.01, ***p < 0.001. (b) Top and bottom 10 distances of words of interest to the fitted decision boundary, out of 261 words defined in previous works (Berger et al. 2021). Red gradients represent placebo non‐responder predictions, whilst blue gradients represent placebo responder predictions. (c) Scatter plot of all sentence clusters found with study 1. Dashed line represents the learned decision boundary. Red gradients represent placebo non‐responder predictions, whilst blue gradients represent placebo responder predictions. Below, the automatically generated cluster description for two placebo non‐responder cluster predictions and two placebo responder cluster predictions. (d) Scatter plot of all sentence clusters found with “r/backpain”. Dashed line represents the learned decision boundary. Red gradients represent placebo non‐responder predictions, whilst blue gradients represent placebo responder predictions. Below, the automatically generated cluster description for two placebo non‐responder cluster predictions and two placebo responder cluster predictions.

To further understand the features selected by the classification model, and the possible redundancy with our previous results, we studied the association between the two selected contextual features (features 1 and 2, mentioned above) from our model and linguistic, demographic, and clinical variables which had been explored in previous works (Vachon‐Presseau et al. 2022; Vachon‐Presseau et al. 2018), as well as with the language features identified in our past work (Berger et al. 2021; Branco, Berger, et al. 2023). Figure 4a shows that the two contextual features do not significantly correlate with any of the confounding variables, nor the selected clinical questionnaire assessments from (Vachon‐Presseau et al. 2018), implying the signal captured by language is not explained by trivial confounds such as verbosity, and captured information not contained in the tested psychometric dimensions assessed here. Moreover, feature 1 significantly correlates with LIWC/leisure (*r* = −0.266; *p* = 0.037) and SD/identity (*r* = 0.281; *p* = 0.027), although these correlations are weak and would not survive multiple comparison correction. In contrast, feature 2 significantly correlates with SD/magnify (*r* = −0.389; *p* = 0.002), SD/fear (*r* = −0.53; *p* = 0.000009), SD/awareness (*r* = −0.404; *p* = 0.001), SD/loss (*r* = −0.477; *p* = 0.00009), and SD/identity (*r* = −0.414; *p* = 0.0009). Thus, it seems that one of the two features (feature 2) picked up by the fine‐tuned Longformer model does indeed map to similar topics as in our previous manuscript and appears to track cognitive‐affective dimensions hypothesized to be important for chronic pain and placebo. Interestingly, the other feature (feature 1) seems to capture mostly new semantic dimensions; to explore these dimensions further, we ran additional post hoc analyses reported below.

### 3.5 Semantic Concept Distribution Over the Fitted Decision Boundary

Figure 4b shows the top 10 positive and negative distances of the 261 words of interest in the first semantic set to the fitted decision boundary (shown in Figure 3c). Like panels C and D of the same figure, the more negative the value (red gradient), the more confident the non‐responder prediction, and the more positive the value (blue gradient), the more confident the responder prediction. Figure 4c shows the complete list of study 1 sentence clusters (each is a semantic concept represented by its sentences), plotted in reference to features 1 and 2. Each cluster is represented by a single dot, coloured according to its distance to the decision boundary. The cluster position and distance to the decision boundary were calculated according to the vector averaging all included sentence feature vectors. Four clusters, two on each side of the decision boundary, were highlighted for illustrative reasons. These are accompanied by the corresponding Llama‐3‐8B‐Instruct short description, for ease of interpretation. Figure 4d shows the same results for the complete list of “r/backpain” sentence clusters. Note that 124 clusters were automatically found in study 1 interviews, whilst 324 were found in “r/backpain”, which is in line with our hypothesis of unconstrained semantic concepts found in an independent sample of chronic back pain texts. These two plots show an overlap in the 2‐feature space, as well as clusters in regions unique either to study 1 semantics or “r/backpain” semantics. Although the contents of only 8 clusters are illustrated here, all clusters, their corresponding clustered sentences, and short descriptions can be found and explored online at www.tinyurl.com/placeboLLM. To assess the quality of the cluster descriptions automatically generated by Llama‐3‐8B‐Instruct, we requested 3 random raters to score their perceived level of fit of 10% of the descriptions and corresponding sentence clusters (10% of study 1 clusters and 10% of “r/backpain” clusters, randomly chosen, for a total of 44 clusters). The raters were independently asked to give their opinion on how much they agreed with the generated description, given the cluster of sentences, on a Likert scale from 0 (“I do not agree at all”) to 7 (“I completely agree”). We found an average perceived level of fit of 6.11 (95% CI [5.83, 6.38]), with a moderate level of average inter‐rater agreement, as indicated by an ICC (2, k) of 0.53 (*p* < 0.001). From this, we conclude that the automatically generated cluster descriptions are, on average, perceived as a good fit for their corresponding cluster sentences. Note these labels are only used for summarization, and the full content of each cluster can be inspected in the link above.

### 3.6 Model Validation on Study 2

**FIGURE 5:**
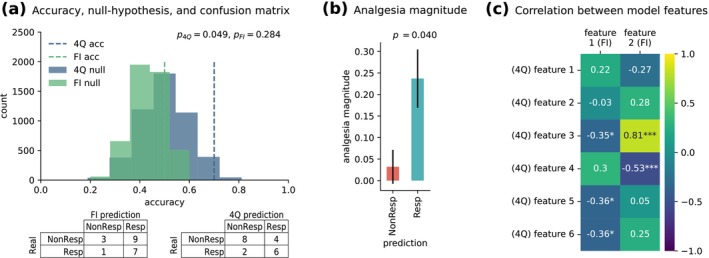
Model validation on study 2. (a) Model validation stage leveraging study 1 interviews for model fitting (single CV with hyperparameter optimization) and study 2 interviews for model testing, comparing two validation approaches. The full interview (FI; i.e., using all 16 topics in study 1 interviews) validation approach had a non‐statistically significant prediction accuracy of 50% (*p* = 0.284). The 4‐question (4Q; using only the 4 matching topics from study 1 interviews) validation approach had a statistically significant prediction accuracy of 70% (*p* = 0.049). Both results are shown in comparison to the corresponding null hypothesis distributions. Below, the corresponding study 2 confusion matrices. (b) Expected analgesia magnitude of predicted placebo treatment outcomes in study 2, based on 4Q model predictions. The difference between the two groups is statistically significant (*p* = 0.040), highlighting that patients predicted a priori as being placebo responders indeed show higher levels of pain relief. (c) Correlation between model features from the FI model (total of 2 significant features) and the 4Q model (total of 6 significant features) shows that while one of the FI features overlaps well with the features from the 4Q model (feature 2), the other FI feature (feature 1) is weakly correlated to features from the 4Q model. In other words, the two models capture a mix of overlapping and unique signals, likely due to differences in the interview length and content and possibly explaining why the FI model may not validate successfully in the study 2; *p < 0.05 and ***p < 0.001.

Thus far, the models discussed above were fitted and validated on study 1 interviews. As shown in Figure 1, these interviews were conducted after treatment. Although we explicitly removed all interview prompts regarding treatment outcomes (see details of our preprocessing methods), and we did not find differences in the predictive language features between the treatment and no‐treatment groups, we acknowledge this limitation on the development study. To overcome this, we attempted to validate this model on an independent sample of patients (study 2). These interviews were conducted before treatment, mimicking the predictive nature of our task, and overcoming the limitations of study 1. The idiosyncrasies of study 2, namely a much shorter interview script (4 out of the 16 original interview topics), and our model's potential dependence on context prompted us to test two different validation approaches: by testing the full model (i.e., trained on the whole text) on study 2, or by instead refitting the best performing model only on the four questions that overlap between the two studies. This was decided a priori. When testing the full interview model from study 1, the model did not validate in this out‐of‐sample dataset (Figure 5a, 50% accuracy; *p* = 0.284). However, when the model is trained on the four overlapping interview topics only, it did validate in study 2 (Figure 5a, 70% accuracy; *p* = 0.049), identifying six latent features with predictive ability. Since these two validation approaches yielded two different models, we examined, post hoc, how the features extracted from both models compare. To do so, we generated a correlation matrix between the two original features, extracted with the full model interview (FI), and the six new features, extracted with the model limited to the 4 overlapping topics (4Q). Figure 5c shows the correlation matrix between features extracted with the two models. Indeed, one of the latent features from the FI model (feature 2) was strongly correlated with features from the 4Q model (strongest correlated feature, *r* = 0.81, *p* < 0.001), suggesting it is indeed tapping into similar latent semantic concepts; in contrast, feature 1 from FI shows only moderate associations with 4Q features (all *rs* < 0.36), hence likely capturing semantic concepts not present when the text is restricted to four questions only. These results are in line with our hypothesis that the full‐interview model would learn to leverage context that would not be available in the test interviews (i.e., study 2), and thus underperform when such data is not available. Finally, in Figure 5c, the difference in analgesia magnitude between predicted placebo responders and non‐responders, according to model 4Q, was statistically significant (independent samples *t*‐test, *p* = 0.040), i.e., individuals predicted as placebo responders did, indeed, show significantly higher levels of pain relief.

## 4 Discussion

In this study, we tested the ability to predict placebo responses in chronic pain patients using contextual features from a language model fine‐tuned to chronic pain texts and assessed the models' ability to identify latent topics that relate to placebo responses. Our results can be summarised in three points. First, semantic features extracted with fine‐tuned language models were able to predict which individuals responded to a placebo. Second, language modelling methods were able to leverage contextual information to predict placebo responses without the need to pre‐select search terms, and while they were able to predict placebo responses in an independent dataset, their prediction ability and generalizability—at least with limited clinical data—is contingent on domain adaptation and similar interview structure. Third, the features extracted from language models, combined with a machine‐learning pipeline, allow for the generation of a decision boundary space for placebo response prediction, thus enabling a more precise interpretation of underlying topics predictive of placebo, and showcasing how open‐ended interviews can be used to generate unique insights into the psychosocial determinants of placebo response.

The machine learning model trained on study 1 data was able to predict which individuals responded to a placebo with a significant classification accuracy of 74% in unseen data, as measured through nested cross‐validation. This finding corroborates our previous results (Berger et al. 2021), which used a fundamentally different language processing pipeline with important conceptual and methodological limitations. Conceptually, our previous work required selecting a series of predefined topics, which constrained the search of semantic topics and potentially excluded novel predictive concepts. Technically, our previous methods treated each word as an independent observation and were unable to disambiguate the meaning of each word given the surrounding context. Since describing pain and related concepts requires translating a subjective multi‐dimensional experience into words, individuals often resort to using metaphorical language and other language tools to convey the significance of the pain experience (Lascaratou 2007) (e.g., stabbing through my leg). Consequently, models that are not able to capture and account for such nuanced semantics may be suboptimal, subject to linguistic biases, and therefore, their interpretability may be limited. In this work, we overcame such limitations by extracting latent semantics from fine‐tuned language models, thus eliminating the need to select a priori topics and making the model completely data‐driven and thus less dependent on—and biased by—the experimenter and the topic selection strategy. We leveraged domain adaptation by fine‐tuning the Longformer model to the back pain domain. Indeed, this model fine‐tuning step improved feature extraction quality, increasing the accuracy of the base model from 67% to 74%, thus showing that fine‐tuning improves the model and that social media data can be used to partially overcome limited access to clinically validated patient pain interviews. We note that the accuracy metrics reported here (74%) underperform the accuracy metrics obtained in our previous work, which obtained 79% out‐of‐sample accuracy, but with important trade‐offs, as will be next discussed. Nonetheless, the reproducibility of our previous findings with a completely different methodology indeed shows that NLP is well‐suited to predict placebo responses—at least in controlled experimental conditions and with similar interviews—and also supports the concept that placebo responses are predictable from biopsychosocial features (Branco, Vachon‐Presseau, et al. 2023; Horing et al. 2014). Importantly, we note that this accuracy and generatability across studies does not imply that those individuals labelled as “placebo responders” would respond to placebo in other contexts or even at a later time within the same context. This represents a key challenge in placebo research: disentangling the relative contributions of stable individual traits (e.g., personality characteristics) and more dynamic states (e.g., state anxiety, momentary expectations, ongoing clinical interactions). While prior work underscores the importance of contextual factors in eliciting placebo responses (Atlas 2021; Horing et al. 2014), there is also evidence that more stable traits, including genetic features, may also contribute to placebo (Branco, Vachon‐Presseau, et al. 2023; Hall et al. 2015). Overall, our findings suggest that the placebo response is a predictable phenomenon shaped by biopsychosocial influences, with language providing a powerful means to characterise responder profiles—though further research is needed to determine whether these reflect stable traits or transient states.

Traditional machine‐learning NLP pipelines use explicit knowledge‐ and domain‐informed feature extraction steps (e.g., by calculating semantic distances to a priori target words/concepts) as anchors for model interpretability (LeCun et al. 2015). Here, instead, we leverage language models–which encode latent semantics and syntax as features in a high‐dimensional space (Devlin et al. 2019)–allowing for a closer inspection of the semantic topics used for prediction. To do so, we first examined the associations between the two latent semantic features picked up by our model with demographic, clinical and psycholinguistic variables, and found no significant associations. We also compared these two novel features to the features extracted in our past work to assess if the signal being captured largely overlapped (i.e., whether the model is predicting from the same signals). One of the semantic features correlated strongly with the language features extracted from our previously published work—more specifically, mapping into semantic dimensions of fear, awareness, and magnification. In contrast, a second significant latent semantic dimension showed weak to null correlations to these established language features, implying that the model was able to capture novel semantic domains that are also predictive of placebo responses.

To further understand our model and which novel signals it is capturing, we extracted embedding representations for 261 placebo‐related words we selected previously (Berger et al. 2021) and calculated their distance to the model's learned decision boundary separating predicted placebo responses from non‐responses, effectively quantifying how each semantic concept would contribute to the placebo response. This approach identified a rich new set of semantic dimensions present throughout the subject interviews that could be used to predict placebo responses, most notably “anxiety”, “avoidance” and “wonder” as being strongly predictive of placebo non‐responders, and “sharing”, “hope”, and “present” as predictive of placebo responders. Next, to take advantage of the ability of our model to provide semantic representations at the sentence level (and with the added benefit of using context to aid in interpretability), we found data‐driven clusters of similar sentences in patient interviews and social media, extracted the same significant latent semantic features, and observed each cluster's distance to the learned decision boundary. With this approach, we found that data‐driven concepts such as regret (i.e., a cluster of similar sentences reliably automatically labelled as “regretful realisation,” such as “I just never thought about it, just never even thought about it,” and “I never thought about that question”) and “actively seeking medical information” were used to predict placebo non‐responders, whilst concepts such as “resignation” and “acceptance” most strongly predicted placebo responders. Importantly, although the interviews excluded explicit questions about the placebo intervention or expectations of improvement, the linguistic patterns identified by these interpretation analyses do not appear to reflect literal references to the treatment or expectations regarding the study. Rather, the predictive features capture latent semantic dimensions in participants' narratives and sometimes literal descriptors of activities, treatments, and others, which are unrelated to the study. Indeed, the clustering analyses revealed overall themes of resignation and acceptance (e.g., “I just live with it”), but also more literal expressions, including mentions of pain persistence (“but the pain is still there”), inactivity (“sitting for long periods of time”) or treatment efficacy of alternative treatments (“yoga definitely helps”) as contributing to the prediction of placebo responses. Taken together, this methodology allowed us to identify several novel concepts not captured by our previous work using simpler language pipelines (Berger et al. 2021). These can be projected in model space to provide novel insights into what drives individuals to respond or not to a placebo pill. All semantic concepts, their distance to the prediction decision boundary, and corresponding text excerpts can be inspected in www.tinyurl.com/placeboLLM.

The placebo effect is widely understood to arise from learning and expectations (Atlas 2021; Colloca and Miller 2011; Rossettini et al. 2020), which may also arise from affective appraisals, that is how individuals interpret, evaluate, and assign meaning to their bodily states and treatment context (Ashar et al. 2017; Koban et al. 2017). From this viewpoint, expectations are not isolated cognitive beliefs but arise from broader processes of appraisal that integrate prior experiences, memories, and self‐referential meaning. It is not clear how these can best be quantified since they reflect the individual's subjective interpretation of, e.g., past events, medical experiences, and even their own view of self. Arguably, narrative language features may provide access to these processes: they capture how individuals narrate their pain and treatment experiences from their own perspective, revealing how they construct and communicate expectations through language. For instance, the predictive linguistic features discussed above, such as hope, acceptance, and resignation, may therefore reflect underlying affective appraisals that shape the likelihood of responding to placebo with other features reflecting expectations more directly (e.g., success in other treatments predicting placebo responses). Naturally, given the uncertainty about the origin of the signals captured in this study, one must be cautious not to reify vector embeddings as direct psychological constructs. Further work is needed to clarify how language can tap into these aspects; nonetheless, our findings show that natural language offers a powerful window into the cognitive–emotional evaluations that underlie placebo responses, possibly bridging linguistic expression with biopsychosocial models of meaning, expectation, and symptom modulation.

Importantly, the interpretation pipeline developed in this study allows for the discovery of concrete and actionable knowledge about the underlying task. For example, our model found that the concept “seeking advice or suggestions for managing backpain” is related to placebo non‐response—for research purposes, this can be used to formulate new hypotheses on the relation between proactivity in learning coping strategies and the placebo response. On the other hand, the ability to predict placebo responses opens up important clinical and research approaches: such models could be integrated into early screening phases of clinical trials to help balance groups or to better interpret placebo‐related variability in outcomes. In clinical settings, similar approaches have the potential to assist clinicians in recognizing when a patient's verbal or emotional expressions signal greater sensitivity to contextual factors, and this information can help tailor communication, enhance treatment engagement, or even use this new knowledge to devise treatment plans, a concept that has been advanced previously (Fadnavis et al. 2022). However, these applications remain speculative at this stage. Importantly, this novel interpretation pipeline is not limited to the placebo response prediction task. It can be similarly applied to any task attempting to learn relations between language and psychological states, and target outcomes.

Finally, an important note is that our model successfully validated in data from an independent study (study 2), but only when the model was trained on overlapping topics across the two studies. Given the differences in length and topic coverage across the two studies, our model did not generalise when trained on a larger topical context and tested on a constrained context. This highlights a key limitation of current NLP techniques, specifically the need for large datasets to generalise effectively when training data differs in content or structure from validation data. Indeed, when comparing the features extracted from the full interview compared to the interview constraining the context, one was largely consistent, while the other was not, suggesting indeed that the model was expecting specific signals that were not present in the independent test sample. Interestingly, this was not an issue with our past results using simpler bag‐of‐words methods, possibly because the large averaging of semantic distances across hundreds of words also averaged out unique properties together with noise, perhaps lending itself to better generalisation with the trade‐off of making each individual feature harder to interpret in isolation. This further underscores the trade‐off between detecting highly specific, context‐dependent signals and maintaining generalizability; greater specificity often comes at the expense of broader applicability. With the growing availability of larger language models and with larger samples, this effect may be mitigated and remains an obstacle to be pursued by future research.

Our study has limitations that need acknowledgment. First, we note that both studies have small sample sizes, which may have limited the ability of LLMs to learn and generalize predictive latent semantic dimensions. However, the replicability of placebo across methods and studies, especially given the variability in methodology across the two studies, points to the generalizability of the methods. Further, due to the limited clinical data, we were not able to fully leverage the Transformer architecture and fine‐tune the classification token, as is typically done for language classification (Vaswani et al. 2017); consequently, we effectively separated the data extraction and data prediction pipelines, which may have further impacted predictive ability. Further, we attempted to overcome the limited clinical data challenge by fine‐tuning our model with social media data for back pain language domain adaptation, but this too entails limitations, in particular inherent biases from social media and Reddit (i.e., the overall population of Reddit is skewed towards male‐identified, under 35 years old from the US), including potential biases in tone and content of such text excerpts. Finally, all semantic concepts predictive of placebo response were captured through data‐driven methods and interpreted using both reverse inference and through correlation with other more well‐established features, and future research is needed to directly assess the contribution of these features in placebo responsiveness, perhaps by testing novel concepts using psychometrics and patient‐reported outcomes.

## Author Contributions

P.B. supervised this work. P.B. and D.A.P.N. performed conceptualization, data curation, formal analysis, investigation, methodology, resources, software, validation, visualisation, original draft writing, review, and editing. D.F. performed original draft writing, review, and editing. S.B. performed conceptualization. S.B., G.C., J.F.‐G., F.N., D.M.M., and A.V.A. performed methodology validation, original draft review and editing, and funding acquisition. All authors discussed the results and commented on the manuscript.

## Funding

This work was supported by National Institutes of Health [R01AR074274] to Apkarian, an unrestricted gift by Google to Branco, by Portuguese national funds through the Fundação para a Ciência e Tecnologia (FCT) [UIDB/50021/2020], and by the Portuguese Recovery and Resilience Plan and Next Generation EU European Funds [C644865762‐00000008] (Accelerat.AI). Diogo A.P. Nunes was supported by a scholarship granted by FCT [2021.06759.BD].

## Conflicts of Interest

The authors declare no conflicts of interest.

## Supporting information


**Data S1:** ejp70184‐sup‐0001‐Supinfo.docx.
